# Diagnostic Performance of Alpha-Fetoprotein, Protein Induced by Vitamin K Absence, Osteopontin, Dickkopf-1 and Its Combinations for Hepatocellular Carcinoma

**DOI:** 10.1371/journal.pone.0151069

**Published:** 2016-03-17

**Authors:** Eun Sun Jang, Sook-Hyang Jeong, Jin-Wook Kim, Yun Suk Choi, Philippe Leissner, Christian Brechot

**Affiliations:** 1 Department of Internal Medicine, Seoul National University College of Medicine, Seoul National University Bundang Hospital, Seoul, Republic of Korea; 2 Medical Diagnostics Discovery Department, bioMérieux, Marcy l’Etoile, France; 3 Mérieux Institute, Lyon, France; Drexel University College of Medicine, UNITED STATES

## Abstract

**Background & Aims:**

Alpha-fetoprotein (AFP) is the most widely used serum biomarker for hepatocellular carcinoma (HCC), despite its limitations. As complementary biomarkers, protein induced by vitamin K absence (PIVKA-II), osteopontin (OPN), and Dickkopf-1 (DKK-1) have been proposed. This study aimed to perform a head-to-head comparison of the diagnostic performance of AFP, PIVKA-II, OPN and DKK-1 as single or in combination to seek the best biomarker or panel, and to investigate the clinical factors affecting their performance.

**Methods:**

Using 401 stored plasma samples obtained from 208 HCC patients and 193 liver cirrhosis control patients, plasma AFP, PIVKA-II, OPN and DKK-1 levels were measured by ELISA, and receiver operating characteristic curve analyses were performed for each biomarker and for every combination of two to four markers.

**Results:**

Of the four biomarkers, AFP showed the highest area under the curve (0.786). The sensitivity and specificity for each single biomarker was 62% and 90.2% (AFP>20 ng/mL), 51.0% and 91.2% (PIVKA-II>10 ng/mL), 46.2% and 80.3% (OPN>100 ng/mL), and 50.0% and 80.8% (DKK-1>500 pg/mL), respectively. Among the combinations of two biomarkers, AFP>20 ng/mL or DKK-1>500 pg/mL showed the best diagnostic performance (sensitivity 78.4%, specificity 72.5%). Triple or quadruple combination did not improve the diagnostic performance further. The patient’s age, etiology and tumor invasiveness of HCC affected the performance of each marker.

**Conclusions:**

AFP was the most useful single biomarker for HCC diagnosis, and the combined measurement of AFP and DKK-1 could maximize the diagnostic yield. Clinical decision should be based on the consideration of various factors affecting the diagnostic performance of each biomarker. Efforts to seek novel HCC biomarkers should be continued.

## Introduction

Hepatocellular carcinoma (HCC) is the third leading cause of cancer-related death worldwide [1]. The serum α-fetoprotein (AFP) is the most widely-used HCC biomarker [2], and many physicians use AFP in clinical practice to diagnose HCC so far [3]. However, the current Western guidelines [4, 5] have excluded AFP measurement for the diagnosis of HCC, because of its limited accuracy in detecting HCC, with a sensitivity of about 60% at a cut-off value of 20 ng/mL [4, 6] and low specificity [7, 8].

To complement the limitations of AFP, the combined measurement of AFP and a protein induced by vitamin K absence (PIVKA-II) [9–11] or other biomarkers such as osteopontin (OPN) or Dickkopf-1 (DKK-1) have been used. OPN, a secreted phosphoprotein that binds to αV-integrin and a cluster of the CD44 family of receptors, showed better sensitivity, specificity, and area under the receiver operating characteristic curve (AUC) than AFP or PIVKA-II [12, 13] for the early diagnosis of HCC. DKK-1, a secretory antagonist of the Wnt signalling pathway, was recently reported as a promising biomarker for HCC, even in AFP-negative patients, and a combination of AFP and DKK-1 measurement showed an improved diagnostic accuracy among HBV infected patients [14].

Despite their success, a head-to-head comparison of diagnostic performance of the four most promising tumor markers for HCC (AFP, PIVKA-II, OPN, and DKK-1) has yet to be reported. The aim of the current study was to determine the diagnostic performance of these biomarkers for the detection of HCC by comparing the sensitivity and specificity of each biomarker alone and in combination among HCC patients and a control group of liver cirrhosis (LC) patients. In addition, clinical factors related to the diagnostic performance of each biomarker were analysed.

## Materials and Methods

### Patients

A total of 401 patients (208 HCC and 193 LC) were enrolled at Seoul National University Bundang Hospital (Seongnam, Republic of Korea) from January 2008 to December 2012. Plasma samples were obtained from newly diagnosed HCC patients without extrahepatic malignancy. HCC was diagnosed based on histological findings or typical imaging characteristics as defined by the Korean Liver Cancer Study Group guidelines, which are similar to the AASLD guidelines [15]. HCC staging was determined using the Barcelona Clinic Liver Cancer (BCLC) staging system [5]. LC was diagnosed by histological examination or clinical findings of portal hypertension [6], and LC patients underwent adequate imaging studies to exclude HCC within 6 months of registration. There was no LC patient who diagnosed HCC within 6 months of enrolment.

For this case-control study, gender and the etiology of liver disease were matched as much as possible in the HCC and the LC groups, although it could not fulfill the 1:1 criteria due to the small number of HCV-related HCC and non-viral LC patients.

This study was conducted according to the principles expressed in the Declaration of Helsinki. Seoul National University Bundang Hospital’s Institutional Review Board which was accredited by the Association for the Accreditation of Human Research Protection Programs (AAHRPP) approved this study (IRB No. #B-1304/197-001 and #B-1307/210-006). All HCC subjects provided written informed consent to participate in this study. Plasma samples from LC patients obtained from a repository that included anonymized clinical data after IRB approval and all data was de-identified prior to analysis.

### Sample storage and assays

A 4 mL peripheral blood sample was collected in an EDTA tube from each patient before the initial treatment for the HCC group or at the time of the clinic visit for the control group. Plasma aliquots were stored at -70°C until measurement.

Plasma AFP, PIVKA-II, OPN and DKK-1 levels were measured for each sample in duplicate by an experienced technician who was blind to the clinical information. Once the frozen samples were thawed, further refreezing was abandoned. AFP was measured using an automated quantitative enzyme linked fluorescent assay (ELFA) with mini-VIDAS^®^ AFP (Biomerieux, Marcy-L’Etoile, France). PIVKA-II (Cusabio Biotech Co., Ltd., Wuhan, China), and both OPN and DKK-1 (R&D Systems, Inc. Minneapolis, MN, USA) were measured using commercially available enzyme-linked immunosorbent assay (ELISA) kits according to the manufacturer’s instructions. Adequate standard curves were generated for each ELISA plate used. If results from duplicate assay showed a difference with more than 10% coefficient variation (CV), repeated duplicate assay was performed, and only reliable results were included for the analysis.

### Statistical analysis

To compare diagnostic performance, receiver operating characteristics (ROC) curves were plotted for each biomarker and for every combination of 2–4 markers. The optimal cut-off value was determined as the level showing the minimum distance to the top-left corner of each ROC curve using a single marker (S1 Table). We defined that each combination was positive (diagnostic for HCC) if the result of any marker in the combination was positive. For example, a positive result for the combination of AFP > 20 ng/mL and DKK > 500 pg/mL meant a sample showed AFP > 20 ng/mL or DKK > 500 pg/mL. Differences between the area under the ROC curve (AUC) of each biomarker for distinguishing between HCC and LC patients and their 95% confidence intervals (CI) were calculated using the DeLong method with paired AUC comparison test by the pROC package in R (R Development Core Team, 2014, http://www.R-project.org).

The sample size needed was calculated by the PASS software (NCSS, Kaysville, UT, USA) using the criteria of 80% power and an alpha of 0.05 for the comparison AUC of AFP, with the assumption that a significantly better marker or marker combination could have an additional 10% AUC increase compared to AFP. All statistical analysis was performed with SPSS for Windows (version 18, SPSS Inc., Chicago, IL, USA) and R software.

## Results

### Patient characteristics and biomarker levels

Among 401 patients (208 HCC and 193 LC), 270 (67.3%) were infected with hepatitis B virus (HBV) and 62 (15.5%) with hepatitis C virus (HCV). Of the 208 HCC cases, 178 (85.6%) had cirrhosis as a background liver disease (Table 1). The BCLC stages of enrolled HCC patients were 0 in 27 (13.0%), A in 70 (33.7%), B in 16 (7.7%), C in 86 (41.3%) and D in 9 (4.3%) (Table 1).

**Table 1:** Clinical characteristics of the study population.

	Total (N = 401)	LC (N = 193)	HCC (N = 208)	p-value
**Age**^1^	59.5 (11.46)	57.85 (10.97)	61.02 (11.71)	0.005
**Gender**, male^2^	278 (69.3)	107 (55.4)	171 (82.2)	<0.001
**BMI**^1^	24.01 (4.08)	24.20 (4.57)	23.83 (3.56)	0.361
**Etiology**^2^				
Alcohol	29 (7.2)	5 (2.6)	24 (11.5)	<0.001
HBV	270 (67.3)	130 (67.4)	140 (67.3)	
HCV	62 (15.5)	41 (21.2)	21 (10.1)	
Cryptogenic	37 (9.2)	17 (8.8)	20 (9.6)	
Others	3 (0.7)	0 (0.0)	3 (1.4)	
**Child-Pugh class**^2^				
A	334 (83.3)	162 (83.9)	172 (82.7)	0.607
B	57 (14.2)	25 (13.0)	32 (15.4)	
C	10 (2.5)	6 (3.1)	4 (1.9)	
**MELD score**^1^	7.41 (5.75)	9.12 (6.03)	5.81 (4.96)	<0.001
**Liver cirrhosis**^2^	371 (92.5)	193 (100.0)	178 (85.6)	<0.001
**Tumor characteristics**^2^				
BCLC stage				
0/ A/ B/ C/ D			27 (13.0)/ 70 (33.7)/ 16 (7.7)/ 86 (41.3)/ 9 (4.3)	
TNM stage				
T1/ 2/ 3a/ 3b/ 4			90 (43.3)/ 46 (22.1)/ 25 (12.0)/ 43 (20.7)/ 4 (1.9)	
N1	-	-	10 (4.8)	
M1	-	-	17 (8.2)	
Diffuse type	-	-	28 (13.5)	
With major PVI	-	-	47 (22.5)	

^1^ mean (SD)

^2^ number (percent in total population)

^3^ number (percent in HBV+ patients)

^4^ number (percent in HCV+ patients)

LC, liver cirrhosis; HCC, hepatocellular carcinoma; BMI, body mass index; HBV, hepatitis B virus; HCV, hepatitis C virus; MELD, model for end-stage liver disease; AFP, alpha-fetoprotein; PIVKA-II, protein induced by vitamin K absence; OPN, osteopontin; DKK-1, Dickkopf -1; BCLC, Barcelona Clinic Liver Cancer; PVI, portal vein invasion

Median plasma AFP, PIVKA-II, OPN and DKK-1 levels were significantly higher in the HCC group than the LC group (AFP, 43.5 (IQR 7.1–839.6) vs. 5.0 (IQR 2.8–9.9) ng/mL; PIVKA-II, 10.7 (IQR 2.5–187.6) vs. 3.0 (IQR 1.2–5.6) ng/mL; OPN, 93.4 (IQR 66.5–197.7) vs. 72.5 (IQR 59.1–91.5) ng/mL; DKK-1, 497.1 (IQR 279.0–782.1) vs. 336.3 (IQR 232.5–446.0) pg/mL; all p-values <0.001, data not shown).

### Comparison of AUC, sensitivity, and specificity of the four biomarkers to distinguish HCC from LC

**Fig 1:**
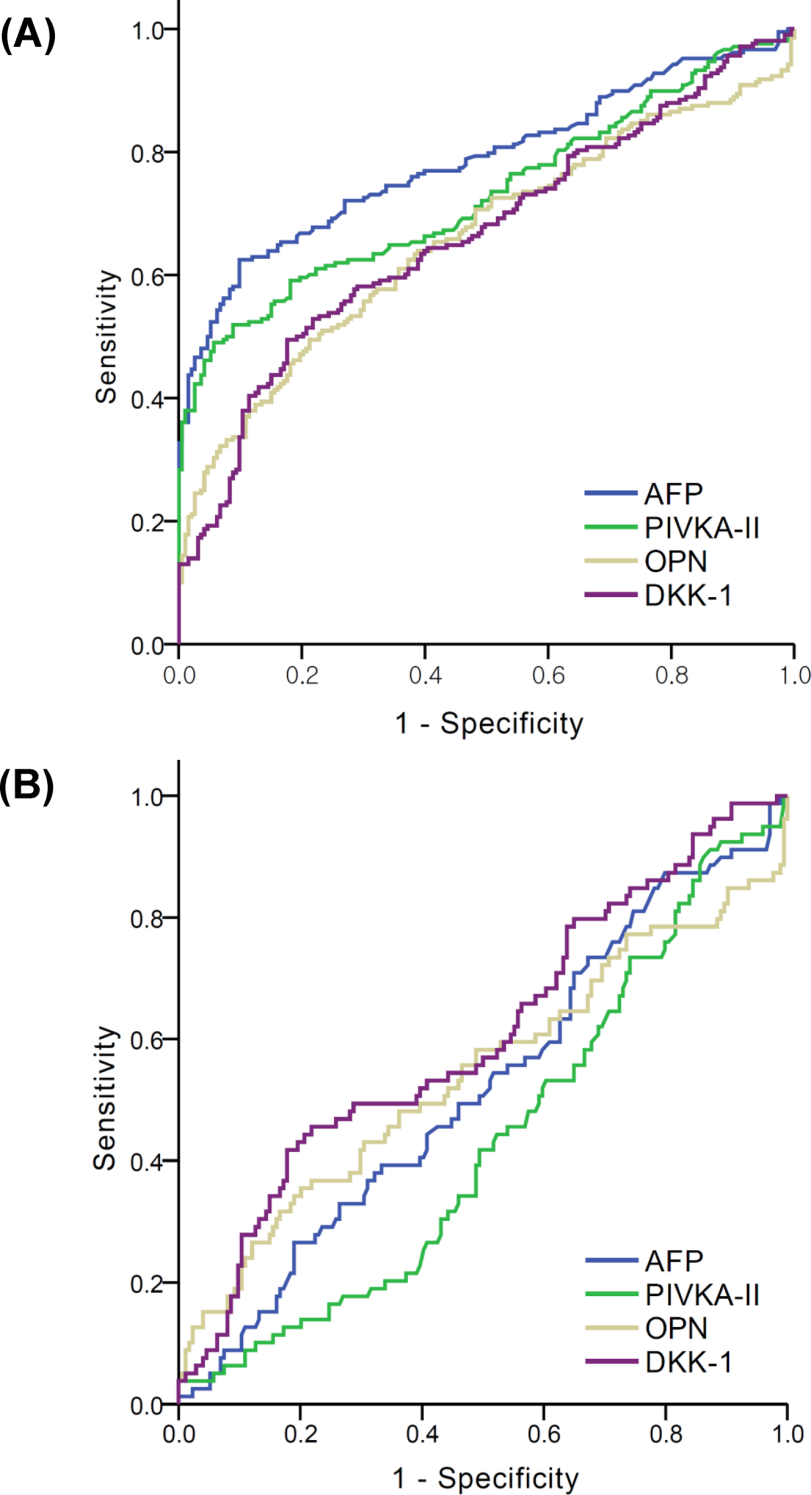
ROC curves of AFP, PIVKA-II, OPN, and DKK-1 for the diagnosis of HCC with LC control. (A) In entire population (B) In the subgroup with AFP levels < 20 ng/mL.

As shown in Fig 1, AFP showed the best AUC (0.786, 95% CI 0.740–0.831) for the diagnosis of HCC, and the AUC for the remaining three biomarkers was 0.729 (95% CI 0.680–0.779) for PIVKA-II, 0.660 (95% CI 0.606–0.713) for OPN, and 0.665 (95% CI 0.612–0.718) for DKK-1. Sn, Sp, negative and positive predictive values (NPV and PPV, respectively) were described with optimal cut-off values for each marker (20 ng/mL for AFP, 10 ng/mL for PIVKA-II, 100 ng/mL for OPN, and 500 pg/mL for DKK-1) in Table 2. As diagnostic markers for HCC, AFP > 20 ng/mL was the most sensitive (62%) with a Sp of 90.2% (Table 2). AFP > 200 ng/mL showed a Sp of 100% and a Sn of 30% (PPV 1.0, NPV 0.58, AUC 0.663 (95% CI 0.610–0.716), data not shown).

**Table 2:** Diagnostic performances of 4 biomarkers for each biomarker and for every combination using 2–4 biomarkers for HCC diagnosis with LC control.

Number of markers	Combination*	Total subjects (N = 401)					
		Sn	Sp	Sn +Sp	PPV	NPV	AUC
Single	AFP	**0.62**	0.902	**1.522**	**0.872**	**0.688**	**0.761**
	PIVKA-II	0.51	0.912	1.422	0.862	0.633	0.711
	OPN	0.462	0.803	1.265	0.716	0.581	0.632
	DKK-1	0.5	0.808	1.308	0.738	0.6	0.654
Double	AFP + PIVKA-II	0.635	**0.876**	**1.511**	**0.846**	0.69	**0.755**
	AFP + OPN	0.75	0.72	1.47	0.743	0.728	0.735
	AFP + DKK-1	**0.784**	0.725	1.509	0.755	**0.757**	**0.755**
	PIVKA-II + OPN	0.678	0.741	1.419	0.738	0.681	0.709
	PIVKA-II + DKK-1	0.736	0.731	1.467	0.746	0.719	0.733
	OPN + DKK-1	0.712	0.632	1.344	0.676	0.67	0.672
Triple	AFP + PIVKA-II + OPN	0.755	**0.705**	1.46	0.734	0.727	0.73
	AFP + PIVKA-II + DKK-1	0.793	0.699	**1.492**	**0.74**	0.758	0.746
	AFP + OPN + DKK-1	**0.846**	0.565	1.411	0.677	**0.773**	0.705
	PIVKA-II + OPN + DKK-1	0.817	0.58	1.397	0.677	0.747	0.699
Quadruple	AFP + PIVKA-II + OPN + DKK-1	**0.851**	**0.549**	**1.4**	**0.67**	**0.774**	**0.7**

* Cut-off values for each marker combination were AFP > 20 ng/mL, PIVKA-II >10 ng/mL, OPN > 100 ng/mL, and DKK-1 > 500 pg/mL.

Numbers in bold are the best sensitivitiy (Sn), specificity (Sp), the sum of Sn+Sp, positive predictive value (PPV), negative predictive value (NPV) and AUC (area under the curve) among various combinations with the same numbers of biomarkers, respectively.

Sn, sensitivitiy; Sp, specificity; PPV, positive predictive value; NPV, negative predictive value; AUC, area under the curve; AFP, alpha-fetoprotein; PIVKA-II, protein induced by vitamin K absence; OPN, osteopontin; DKK-1, Dickkopf -1

When we tested the diagnostic accuracy of combinations with 2 markers, AFP > 20 ng/mL combined with DKK > 500 pg/mL showed an increased Sn (78.4%), but a decreased Sp (72.5%) as compared to AFP alone. The most specific combination using 2 markers was AFP > 20 ng/mL combined with PIVKA-II > 10 ng/mL (Sp of 87.6%, Sn of 63.5%), showing the highest sum of Sn and Sp among all the 2-marker combinations.

Regarding triple marker combinations, the ‘AFP > 20 ng/mL or OPN > 100 ng/mL or DKK-1 > 500 pg/mL’ combination showed the best Sn (84.6%) at the cost of a lower Sp (56.5%). Even with four biomarkers (AFP, OPN, DKK-1 and PIVKA-II), the Sn (85.1%) and Sp (54.9%) were not improved.

### Comparison of AUC, sensitivity, and specificity for distinguishing HCC from LC in the subgroup of HCC with a low AFP level (<20 ng/mL)

In the present study, 79 (37.8%) HCC and 174 (90.1%) LC patients showed low (<20 ng/mL) plasma AFP levels. Thus, we evaluated how many HCC cases in this subgroup could be diagnosed by using other biomarkers.

As a single marker, DKK-1 > 500 pg/mL showed the best AUC (0.617, 95% CI 0.540–0.695), however, the Sn was only 43%. Of the two-marker combinations, DKK-1 > 500 pg/mL combined with OPN > 100 ng/mL resulted in a HCC diagnosis in 59.5% of the low-AFP subgroup. Adding PIVKA-II to the DKK-1 and OPN combination did not improve the Sn (60.8%) for the diagnosis of HCC (Table 3).

**Table 3:** Diagnostic performances of 4 biomarkers for each biomarker and for every combination using 2–4 biomarkers for HCC diagnosis with LC control in subgroup showing a low AFP level (<20 ng/mL).

Number of markers	Combination*	Subgroup showing AFP<20 ng/mL (N = 253)					
		Sn	Sp	Sn +Sp	PPV	NPV	AUC
Single	AFP	-	-	-	-	-	-
	PIVKA-II	0.038	**0.971**	1.009	0.375	0.69	0.505
	OPN	0.342	0.799	1.141	0.435	0.728	0.57
	DKK-1	**0.43**	0.805	**1.235**	**0.5**	**0.757**	**0.617**
Double	AFP + PIVKA-II	-	-	-	-	-	-
	AFP + OPN	-	-	-	-	-	-
	AFP + DKK-1	-	-	-	-	-	-
	PIVKA-II + OPN	0.354	**0.782**	1.136	0.424	0.727	0.568
	PIVKA-II + DKK-1	0.456	0.776	**1.232**	**0.48**	0.758	**0.616**
	OPN + DKK-1	**0.595**	0.626	1.221	0.42	**0.773**	0.611
Triple	AFP + PIVKA-II + OPN	-	-	-	-	-	-
	AFP + PIVKA-II + DKK-1	-	-	-	-	-	-
	AFP + OPN + DKK-1	-	-	-	-	-	-
	PIVKA-II + OPN + DKK-1	**0.608**	**0.609**	**1.217**	**0.414**	**0.777**	**0.608**
Quadruple	AFP + PIVKA-II + OPN + DKK-1	-	-	-	-	-	-

* Cut-off values for each marker combination were AFP > 20 ng/mL, PIVKA-II >10 ng/mL, OPN > 100 ng/mL, and DKK-1 > 500 pg/mL.

Numbers in bold are the best sensitivitiy (Sn), specificity (Sp), the sum of Sn+Sp, positive predictive value (PPV), negative predictive value (NPV) and AUC (area under the curve) among various combinations with the same numbers of biomarkers, respectively.

Sn, sensitivitiy; Sp, specificity; PPV, positive predictive value; NPV, negative predictive value; AUC, area under the curve; AFP, alpha-fetoprotein; PIVKA-II, protein induced by vitamin K absence; OPN, osteopontin; DKK-1, Dickkopf -1

### Comparison of AUC, sensitivity, and specificity to distinguish early stage HCC from LC patients

**Fig 2:**
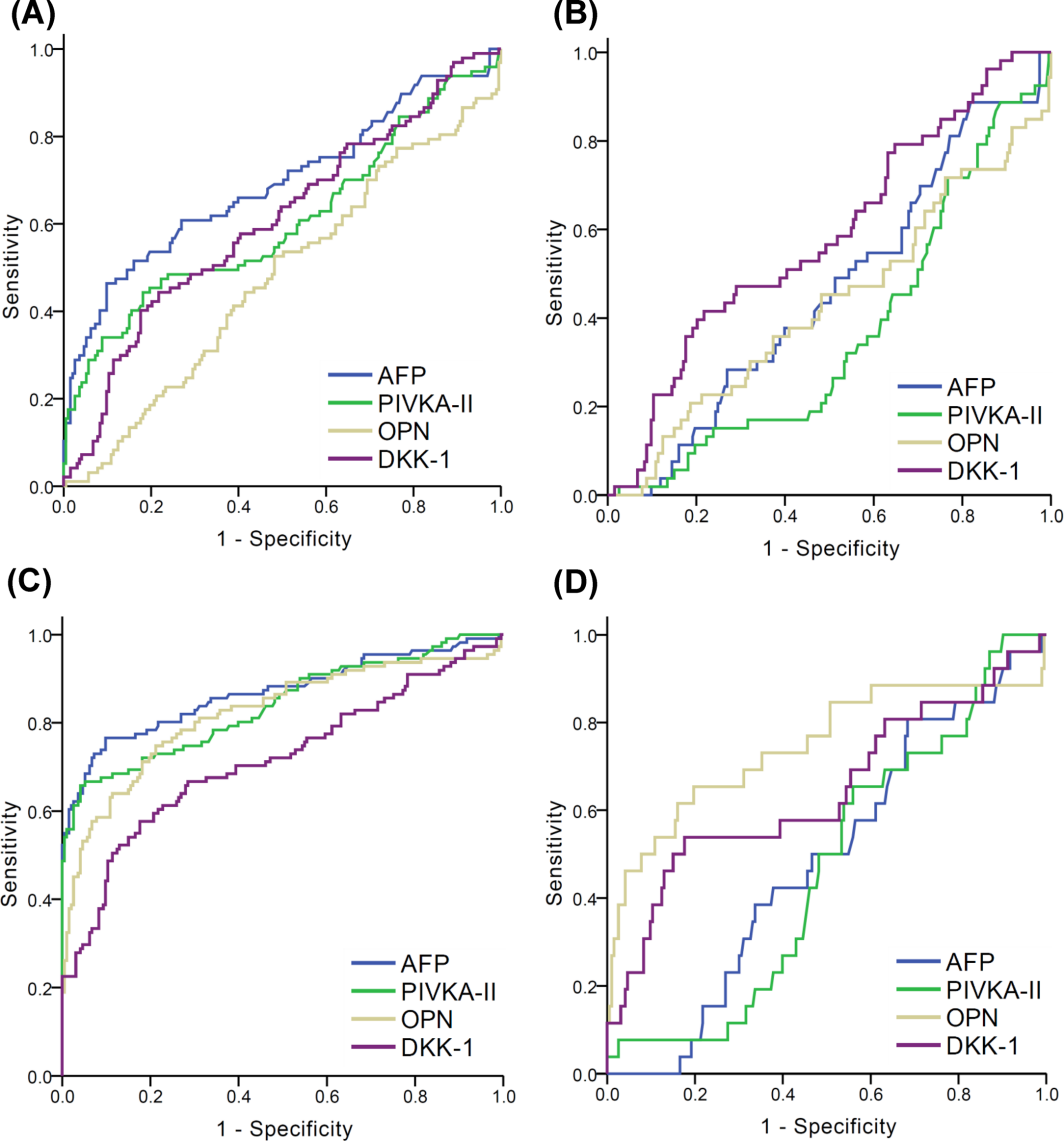
ROC curves of AFP, PIVKA-II, OPN, and DKK-1 for the diagnosis of HCC with LC control in subgroups categorized by BCLC stage and AFP levels. (A) In the subgroup with BCLC 0/A (B) In the subgroup with BCLC 0/A and with plasma AFP level < 20 ng/mL (C) In the subgroup with BCLC B/C/D (D) In the subgroup with BCLC B/C/D and with plasma AFP level < 20 ng/mL. The AUC values are presented in S2 Table.

In this study, 46.6% (97/208) of HCC patients were diagnosed at an early stage (BCLC 0 or A). As a single marker for early HCC diagnosis, AFP was most sensitive and showed the best sum of Sn and Sp (Sn 0.454, Sp 0.902, Table 4). Although PIVKA-II was most specific for the diagnosis of early HCC, the Sn was only 0.32, which was lower than it of DKK-1 (Sn 0.412, Table 4). As shown in Fig 2A, the AUC for AFP (0.691, 95% CI 0.621–0.761, S2 Table), PIVKA-II (0.604, 95% CI 0.530–0.679, S2 Table) and DKK-1 (0.608, 95% CI 0.538–0.678, S2 Table) in patients with BCLC stage 0 or A, which did not show significant difference among the 4 biomarkers. In combination, double markers (AFP > 20 ng/mL or DKK-1 > 500 pg/mL) and triple markers (AFP > 20 ng/mL or PIVKA-II > 10 ng/mL or DKK-1 > 500 pg/mL) showed an AUC of 0.693 (with Sn 66%) and 0.685 (with Sn 67%), respectively (Table 4).

**Table 4:** Diagnostic performances of 4 biomarkers for each biomarker and for every combination using 2–4 biomarkers for early HCC (BCLC stage 0/A) diagnosis with LC control.

Number of markers	Combination*	Subgroup with early stage HCC (BCLC stage 0/A) (N = 290)					
		Sn	Sp	Sn +Sp	PPV	NPV	AUC
Single	AFP	**0.454**	0.902	**1.356**	**0.698**	**0.767**	**0.678**
	PIVKA-II	0.32	**0.912**	1.232	0.646	0.727	0.616
	OPN	0.175	0.803	0.978	0.309	0.66	0.489
	DKK-1	0.412	0.808	1.22	0.519	0.732	0.61
Double	AFP + PIVKA-II	0.464	**0.876**	1.34	**0.652**	0.765	0.67
	AFP + OPN	0.567	0.72	1.287	0.505	0.768	0.644
	AFP + DKK-1	**0.66**	0.725	**1.385**	0.547	**0.809**	**0.693**
	PIVKA-II + OPN	0.464	0.741	1.205	0.474	0.733	0.602
	PIVKA-II + DKK-1	0.588	0.731	1.319	0.523	0.779	0.659
	OPN + DKK-1	0.515	0.632	1.147	0.413	0.722	0.574
Triple	AFP + PIVKA-II + OPN	0.577	**0.705**	1.282	0.496	0.768	0.641
	AFP + PIVKA-II + DKK-1	0.67	0.699	**1.369**	**0.528**	**0.808**	**0.685**
	AFP + OPN + DKK-1	**0.722**	0.565	1.287	0.455	0.801	0.643
	PIVKA-II + OPN + DKK-1	0.67	0.58	1.25	0.445	0.778	0.625
Quadruple	AFP + PIVKA-II + OPN + DKK-1	0.732	0.549	1.281	0.449	0.803	0.641

* Cut-off values for each marker combination were AFP > 20 ng/mL, PIVKA-II >10 ng/mL, OPN > 100 ng/mL, and DKK-1 > 500 pg/mL.

Numbers in bold are the best sensitivitiy (Sn), specificity (Sp), the sum of Sn+Sp, positive predictive value (PPV), negative predictive value (NPV) and AUC (area under the curve) among various combinations with the same numbers of biomarkers, respectively.

Sn, sensitivitiy; Sp, specificity; PPV, positive predictive value; NPV, negative predictive value; AUC, area under the curve; AFP, alpha-fetoprotein; PIVKA-II, protein induced by vitamin K absence; OPN, osteopontin; DKK-1, Dickkopf -1

### Clinical determinants for the diagnostic performance of the four biomarkers for HCC

**Fig 3:**
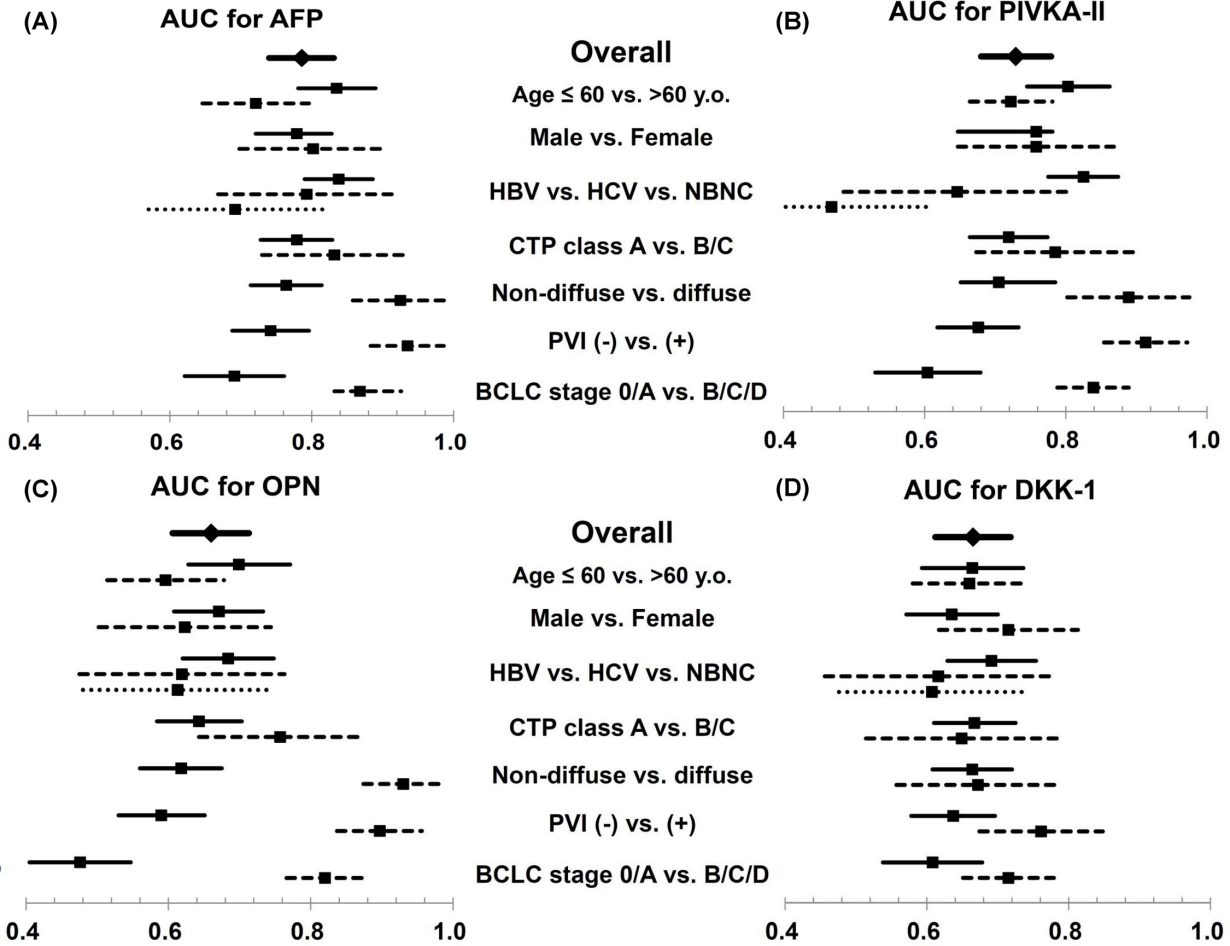
AUCs (with 95% confidence interval) for HCC diagnosis using AFP, PIVKA-II, OPN, and DKK-1 in subgroups categorized by clinical and tumor characteristics. AUCs for HCC diagnosis using AFP (A), PIVKA-II (B), OPN (C), and DKK-1 (D) were schematized to compare the effect of clinical and tumor factors on each biomarker’s diagnostic performance. The diamonds and solid bars represent the AUC and 95% CI of each marker in the total population. The squares and solid bars are the AUC and 95% CI of the first subgroup in each category (Age ≤ 60-years-old, male, HBV, CTP class A, non-diffuse HCC, PVI (-), and BCLC stage 0/A, respectively). The squares and short lined bars denote the AUC and 95% CI of the second subgroup in each category (Age > 60-years-old, female, HCV, CTP class B/C, diffuse HCC, PVI (+), and BCLC stage B/C/D, respectively). The squares and dotted bars are the AUC and 95% CI of the third subgroup in the each category (NBNC). AUC values were not obtained from multivariable analysis. Detailed AUC values with 95% CI and a direct comparison between the AUC of the four markers are presented in S2 Table.

To demonstrate the clinical conditions related to favourable diagnostic performance of each biomarker, the AUC for AFP, PIVKA-II, OPN, and DKK-1 were compared in various subgroup analyses (Fig 3 and S2 Table). The AUCs for AFP and PIVKA-II were significantly lower in the old age (≥ 60 years old) group (p = 0.016 for AFP, Fig 3A; p<0.001 for PIVKA-II, Fig 3B). Tumor invasiveness also affected the diagnostic performance of AFP, PIVKA-II, and OPN, which had higher AUCs in patients with diffuse-type HCC than in those with non-diffuse HCC. However, the AUC for DKK-1 was not affected by the diffuse type of HCC.

The diagnostic yield of the four markers tended to differ depending on the etiology of the underlying liver disease (Fig 3 and S2 Table). Among patients with HBV, AFP (0.838, 95% CI 0.790–0.886), and PIVKA-II (0.825, 95% CI 0.775–0.874) showed a higher AUC compared with OPN (0.684, 95% CI 0.620–0.748) or DKK-1 (0.691, 95% CI 0.629–0.754). In patients with HCV or non-viral liver disease, the AUC for AFP was significantly lower (0.793, 95% CI 0.668–0.918) than that in the patients with HBV (Fig 3 and S2 Table).

## Discussion

By performing a head-to-head comparison of the four diagnostic markers of HCC for the first time, the present study demonstrated that AFP remained the best single marker, and the combined use of AFP with PIVKA-II or DKK-1 had the best diagnostic performance (with a sum of Sn and Sp > 1.5) compared to all other possible combinations of the four biomarkers, even in early stage HCC. Triple or quadruple marker panels did not improve the diagnostic yield compared to the best results obtained from using two markers. In a subgroup with AFP < 20 ng/mL, which accounted for 38% of the study population, DKK-1 showed the best Sn and the best AUC as a single marker. Interestingly, each biomarker had different clinical factors affecting its diagnostic performance, including age, the etiology of liver disease, and the tumor invasiveness.

The most compelling finding of the present study was the demonstration of the comparative diagnostic performance of all possible combinations of the four biomarkers. Because the inclusion of more biomarkers resulted in increased Sn at the cost of decreased Sp [16], the diagnostic yield for HCC did not increase as the number of markers increased (Tables 2, 3 and 4). Since the clinical utility of a biomarker panel should take cost-effectiveness into account [17], additional studies are needed to determine the proper number of combined markers.

In this study, the control group consisted of cirrhotic patients rather than chronic hepatitis patients or healthy people, because most HCC patients have underlying LC, as shown in this study (85.9% of HCC patients had LC). Therefore, the LC control group provides a more stringent and practical comparison for the performance of HCC diagnostic biomarkers. However, using this control group could lower the overall diagnostic yield of the four biomarkers as compared to previous reports [14, 18–22]. As previously documented[10, 12, 14, 23, 24], the diagnostic yield of AFP for HCC is significantly lower when using a control group including more advanced liver disease patients [10, 24], which is comparable to this study. A recent meta-analysis reported the Sn, Sp, and AUC for DKK-1 as 0.65, 0.94 and 0.84, respectively [21], which were superior to results in the present study. However, only two studies using LC controls [14, 25] were included in the meta-analysis, and it may have been biased due to the single large scale study which reported a good AUC (0.858) in LC patients [14]. In contrast, Yang *et al*. [25] showed that the AUC (0.717) for DKK-1 for HCC diagnosis in cirrhotic controls was lower than the AUC (0.877) in their total population of controls including non-cirrhotic chronic hepatitis, benign liver tumor patients and healthy individuals. It was compatible with our result (0.665, 95% CI 0.612–0.718). The characteristics of the cases and the controls profoundly affect the result of biomarker studies, so that a head-to-head comparison of those biomarkers in the same population can reveal their performance more objectively.

The cut-off values for each marker were fixed based on ROC curves to focus on the direct comparison of the four markers in this study (S1 Table). Although the statistically best prediction models for HCC diagnosis could be made by many kinds of logistic regressions [23, 26], these models are hardly applicable when making quick decisions in clinical practice. For example, the best double marker model, with the best sum of Sn and Sp, was ‘AFP > 12.8 ng/ml or DKK > 491.2 pg/mL’. However, the best triple marker model was ‘AFP > 22.2 ng/mL or OPN > 176.8 ng/mL or DKK > 498.1 pg/mL’ (data not shown). Therefore, confusion can result from the changing cut-off values for biomarkers depending on their particular combination. Thus, the authors decided to use the best fixed cut-off value for each marker based on ROC curves to focus on the direct comparison of 4 markers in real-world practice.

Because many factors affecting the diagnostic performance of biomarker should be considered individually in clinical practice, we documented the AUC of all diagnostic markers in various subgroups according to clinical factors in this study (Fig 3). The etiology of the underlying liver disease was considered as a confounding factor for the diagnostic performance of the HCC biomarkers [6, 23]. Marrero *et al*. reported that the AUC for PIVKA-II was better in patients with viral etiology as compared to those with non-viral etiology, whereas AFP was not affected by etiology [6]. In the present study, both PIVKA-II and AFP showed significantly higher AUC in patients with viral etiology as compared to those with non-viral etiology. Moreover, the AUC for AFP in the HBV-infected subgroup showed a better value (0.838, 95% CI 0.790–0.886) than that reported in previous studies (0.69–0.74), which were mostly reported in a pre-antiviral therapy era [6, 12, 13]. This enhanced diagnostic performance of AFP was confirmed in a recent study following the adoption of potent anti-HBV drugs [27]. In our study population, 42.6% of HBV infected LC or HCC patients were treated with antiviral agents, and 72.2% of them showed complete virologic response. With the control of viral replication, the false positive rate of AFP could be minimized and expected to be a more specific biomarker for HCC [28].

In the present study, various subgroup analyses for comparing ROC curves of the four markers were performed despite the possibility of type I error inflation [29]. Although these explorative analyses were not multivariable, we demonstrated that the clinical factors affecting biomarker performance were age, gender, etiology, and the tumor stage. However, the subgroup with HBV had fewer subjects aged > 60 years old (27% in the HBV+ group vs. 75.8% in the HCV+ group vs. 85.5% in the non-viral group, p<0.001, data not shown), suggesting interaction among clinical factors such as etiology and age. We presumed that the trend for the diagnostic performance of each marker did not change in subsequent multi-group analysis considering the interaction with age, HBV-etiology, and tumor stage (data not shown).

As shown in Table 2, DKK-1 was the most sensitive marker for HCC in patients with AFP < 20 ng/mL. In most previous studies, DKK-1 had been demonstrated as a diagnostic marker for early HCC [21]. Although all marker levels were significantly higher in those with advanced HCC, AUC for DKK-1 was lowest among 4 markers in the diffuse type (0.672, 95% CI 0.557–0.786), with PVI (0.761, 95% CI 0.674–0.848), and BCLC B/C/D (0.715, 95% CI 0.651–0.779, Fig 3 and S2 Table). Instead, DKK-1 showed the highest AUC in AFP < 20 ng/mL subgroup with non-diffuse HCC (0.600, 95% CI 0.521–0.678) as well as early BCLC stage (0.586, 95% CI 0.499–0.673, data not shown). Since most advanced HCC could be diagnosed with AFP only, DKK-1 had expected to play a supplementary role for AFP in the setting of early HCC diagnosis.

A limitation of our study was that the samples were obtained at single center, and thus our results require external validation. Nonetheless, internal validation with repeated experiments was performed strictly. Repeated freezing and thawing of plasma was avoided, and in the repeated experiments for samples with high CV, new aliquot of stored samples were used. Another limitation was that we used plasma rather than serum for the measurement of biomarkers due to sample availability, so a direct comparison of DKK-1 levels in this study with those from studies using serum was not possible. According to the product sheet of the kit used in the present study, the mean level of DKK-1 in 36 healthy volunteers was about fourfold in plasma compared to that in serum. Finally, this study aimed to compare clinical utility of 4 biomarkers to diagnose HCC with easy-to-use cut-off values, not to validate the biomarkers as surveillance tool, although the design of the present study was similar to a phase 2 trial for developing early cancer biomarkers [30]. Thus, the enrolled HCC patients were heterogeneous regarding to stages, etiology, and the underlying liver status which reflecting real-world practice. Serial samples following the progression from cirrhosis to HCC will be needed to search for suitable biomarkers for early HCC detection.

In conclusion, AFP is still the most valuable tool for the diagnosis of HCC, as shown by direct comparative analyses of AFP, PIVKA-II, OPN and DKK-1, especially in a HBV-predominant HCC population. DKK-1 seems to be a promising complementary marker in conjunction with AFP, especially in early stage HCC patients whose AFP level is < 20 ng/mL. Clinical decision should be based on the consideration of various factors affecting the diagnostic performance of each biomarker. Prospective validation studies to establish the most efficient and cost-effective marker panel are warranted, and efforts to search for novel, better performing biomarkers should be continued.

## Supporting Information

S1 Table Areas under the receiver operating characteristic curve (with 95% confidence interval) for the HCC diagnosis of AFP, PIVKA-II, OPN and DKK-1 with various cut-off values (DOCX)

S2 Table Areas under the receiver operating characteristic curve (with 95% confidence interval) for the HCC diagnosis of AFP, PIVKA-II, OPN and DKK-1 in the subgroup categorized by clinical and tumoral characteristics (DOCX)

## Abbreviations

AFP: Alpha-fetoprotein
HCC: hepatocellular carcinoma
PIVKA-II: protein induced by vitamin K absence
OPN: osteopontin
DKK-1: Dickkopf-1
AUC: area under the receiver operating characteristic curve
USG: ultrasonography
AASLD: American Association of the Liver Disease
LC: liver cirrhosis
BCLC: Barcelona Clinic Liver Cancer
ROC: receiver operating characteristics
Sn: sensitivity
Sp: specificity
NPV: negative predictive value
PPV: positive predictive value
CI: confidence interval
HBV: hepatitis B virus
HCV: hepatitis C virus
PVI: portal vein invasion
