# Supplementary material for: Diagnostic Performance of Alpha-Fetoprotein, Protein Induced by Vitamin K Absence, Osteopontin, Dickkopf-1 and Its Combinations for Hepatocellular Carcinoma
Source: PLoS One. 2016 Mar 17;11(3):e0151069. doi: 10.1371/journal.pone.0151069 (PMC4795737; doi:10.1371/journal.pone.0151069)
Supplement: S1 Table — (DOCX) [file pone.0151069.s001.docx]

**S1 Table. Areas under the receiver operating characteristic curve (with 95% confidence interval) for the HCC diagnosis of AFP, PIVKA-II, OPN and DKK-1 with various cut-off values**

| Marker | Cut-off | AUC | 95% CI |
| --- | --- | --- | --- |
| AFP (ng/mL) | > 200 | 0.663 | 0.610-0.716 |
|  | > 100 | 0.689 | 0.637-0.741 |
|  | > 50 | 0.722 | 0.672-0.772 |
|  | **> 20** | **0.761** | **0.713-0.809** |
|  | > 10 | 0.722 | 0.671-0.772 |
|  | > 5 | 0.645 | 0.591-0.700 |
| PIVKA-II (ng/mL) | > 1000 | 0.572 | 0.516-0.628 |
|  | > 150 | 0.632 | 0.578-0.686 |
|  | > 100 | 0.642 | 0.588-0.695 |
|  | > 50 | 0.675 | 0.623-0.728 |
|  | > 20 | 0.696 | 0.644-0.747 |
|  | **> 10** | **0.711** | **0.660-0.762** |
|  | > 5 | 0.667 | 0.614-0.721 |
| OPN (ng/mL) | > 1000 | 0.511 | 0.448-0.574 |
|  | > 156 | 0.572 | 0.209-0.636 |
|  | **> 100** | **0.577** | **0.512-0.639** |
|  | > 91 | 0.574 | 0.511-0.637 |
|  | > 50 | 0.484 | 0.421-0.547 |
|  | > 25 | 0.492 | 0.529-0.555 |
|  | > 10 | 0.489 | 0.426-0.552 |
|  | > 9.3 | 0.493 | 0.430-0.556 |
| DKK-1 (pg/mL) | > 1000 | 0.527 | 0.464-0.591 |
|  | **> 500** | **0.626** | **0.564-0.687** |
|  | > 300 | 0.562 | 0.500-0.625 |
|  | > 200 | 0.522 | 0.459-0.584 |
|  | > 100 | 0.513 | 0.450-0.575 |
|  | > 50 | 0.499 | 0.436-0.562 |
|  | > 20 | 0.500 | 0.437-0.563 |
|  | > 5 | 0.500 | 0.437-0.563 |

Numbers in bold are the selected cut-off values for each marker in the present study.

AUC, area under the curve; CI, Confidence Interval; AFP, alpha-fetoprotein; PIVKA-II, protein induced by vitamin K absence; OPN, osteopontin; DKK-1, Dickkopf -1
