# Supplementary material for: Diagnostic Performance of Alpha-Fetoprotein, Protein Induced by Vitamin K Absence, Osteopontin, Dickkopf-1 and Its Combinations for Hepatocellular Carcinoma
Source: PLoS One. 2016 Mar 17;11(3):e0151069. doi: 10.1371/journal.pone.0151069 (PMC4795737; doi:10.1371/journal.pone.0151069)
Supplement: S2 Table — (DOCX) [file pone.0151069.s002.docx]

**S2 Table.** **Areas under the receiver operating characteristic curve (with 95% confidence interval) for the HCC diagnosis of AFP, PIVKA-II, OPN and DKK-1 in the subgroup categorized by clinical and tumoral characteristics.**

| Caterogy | | Criteria | Sub-total | LC | HCC | AFP | PIVKA-II | OPN | DKK-1 | *p*-value | | | | | |
| --- | --- | --- | --- | --- | --- | --- | --- | --- | --- | --- | --- | --- | --- | --- | --- |
|  |  |  |  |  |  |  |  |  |  | AFP vs. PIVKA-II | AFP vs. OPN | AFP vs. DKK-1 | PIVKA-II vs. OPN | PIVKA-II vs. DKK-1 | OPN vs. DKK-1 |
| All subjects | | | 401 | 193 | 208 | 0.786 (0.740-0.831) | 0.729 (0.680-0.779) | 0.66 (0.606-0.713) | 0.665 (0.612-0.718) | **0.040** | 0.656 | 0.117 | **0.041** | **0.0007** | 0.288 |
| Age group | Age ≤ 60 years old | | 222 | 115 | 107 | 0.835 (0.781-0.890) | 0.803 (0.745-0.862) | 0.699 (0.628-0.771) | 0.664 (0.593-0.736) | 0.339 | 0.725 | 0.847 | 0.711 | 0.595 | 0.912 |
|  | Age > 60 years old | | 179 | 78 | 101 | **0.721 (0.646-0.796)*** | **0.628 (0.546-0.710)*** | 0.596 (0.514-0.678) | 0.660 (0.580-0.740) | 0.050 | 0.321 | **0.008** | **0.008** | **<0.001** | 0.165 |
| Gender | Male | | 278 | 107 | 171 | 0.774 (0.721-0.828) | 0.722 (0.664-0.781) | 0.671 (0.608-0.733) | 0.635 (0.571-0.700) | 0.128 | 0.740 | 0.690 | 0.141 | 0.117 | 0.924 |
|  | Female | | 123 | 86 | 37 | 0.802 (0.698-0.906) | 0.758 (0.647-0.868) | 0.623 (0.502-0.744) | 0.715 (0.617-0.813) | 0.447 | 0.379 | **0.006** | 0.087 | **<0.001** | 0.251 |
| Etiology | HBV | | 270 | 130 | 140 | 0.838 (0.790-0.886) | 0.825 (0.775-0.874) | 0.684 (0.620-0.748) | 0.691 (0.629-0.754) | 0.856 | 0.512 | 0.236 | 0.673 | 0.154 | 0.113 |
|  | HCV | | 62 | 41 | 21 | 0.793 (0.668-0.918) | **0.646 (0.485-0.807)*** | 0.619 (0.475-0.763) | 0.616 (0.456-0.775) | 0.632 | 0.220 | 0.673 | **0.046** | 0.379 | 0.251 |
|  | Not-viral cause (alcohol, cryptogenic, others) | | 69 | 22 | 47 | **0.692 (0.570-0.815)*** | **0.468 (0.333-0.603)*** | 0.613 (0.480-0.747) | 0.607 (0.476-0.738) | **<0.001** | 0.893 | 0.863 | **<0.001** | **<0.001** | 0.766 |
| Antiviral treatment (in HBV+ patients) | On anti-HBV Treatment | | 115 | 71 | 44 | 0.746 (0.640-0.851) | 0.711 (0.607-0.815) | 0.478 (0.364-0.592) | 0.633 (0.528-0.737) | 0.781 | 0.378 | 0.241 | 0.574 | 0.118 | 0.062 |
|  | Treatment naïve including inactive carriers | | 155 | 59 | 96 | **0.873 (0.820-0.926)*** | **0.872 (0.818-0.925)*** | **0.783 (0.710-0.856)*** | 0.733 (0.655-0.810) | 0.781 | 0.711 | 0.762 | 0.915 | 0.585 | 0.587 |
| ALT | ALT ≤ UNL (40 IU/L) | | 231 | 120 | 111 | 0.742 (0.675-0.808) | 0.690 (0.620-0.759) | 0.589 (0.514-0.664) | 0.652 (0.581-0.723) | 0.168 | 0.771 | 0.211 | 0.400 | **0.012** | 0.173 |
|  | ALT > UNL (40 IU/L) | | 170 | 73 | 97 | **0.835 (0.776-0.895)*** | 0.769 (0.699-0.839) | **0.747 (0.675-0.820)*** | 0.678 (0.597-0.758) | **0.011** | 0.341 | 0.467 | **0.007** | **0.007** | 0.785 |
| Child-Pugh class | A | | 334 | 162 | 172 | 0.779 (0.728-0.829) | 0.719 (0.664-0.774) | 0.643 (0.584-0.703) | 0.667 (0.610-0.725) | **0.042** | 0.91 | 0.245 | 0.127 | **0.003** | 0.233 |
|  | B/C | | 67 | 31 | 36 | 0.832 (0.730-0.934) | 0.785 (0.673-0.896) | 0.757 (0.643-0.871) | 0.649 (0.514-0.783) | 0.643 | 0.115 | 0.226 | **0.05** | 0.118 | 0.634 |
| Tumor type | Non-diffuse type | | 373 | 193 | 180 | 0.764 (0.714-0.814) | 0.705 (0.651-0.758) | 0.618 (0.560-0.675) | 0.664 (0.608-0.720) | 0.057 | 0.969 | 0.136 | 0.118 | **0.001** | 0.177 |
|  | Diffuse type | | 221 | 193 | 28 | **0.925 (0.858-0.992)*** | **0.889 (0.802-0.975)*** | **0.930 (0.874-0.987)*** | 0.672 (0.557-0.786) | 0.19 | **0.002** | 0.449 | **<0.001** | 0.187 | 0.073 |
| Tumor type | PVI (-) | | 354 | 193 | 161 | 0.742 (0.688-0.796) | 0.676 (0.618-0.733) | 0.590 (0.530-0.651) | 0.637 (0.578-0.696) | **0.045** | 0.944 | 0.187 | 0.125 | **0.002** | 0.204 |
|  | PVI (+) | | 240 | 193 | 47 | **0.935 (0.883-0.986)*** | **0.913 (0.854-0.972)*** | **0.897 (0.837-0.956)*** | **0.761 (0.674-0.848)*** | 0.519 | **<0.001** | **0.012** | **<0.001** | **0.028** | 0.191 |
| T stage | T1 | | 283 | 193 | 90 | 0.681 (0.608-0.754) | 0.602 (0.525-0.679) | 0.551 (0.476-0.626) | 0.606 (0.533-0.679) | **0.031** | 0.855 | 0.187 | 0.067 | **0.001** | 0.299 |
|  | T2/3/4 | | 311 | 193 | 118 | **0.865 (0.819-0.912)*** | **0.827 (0.775-0.878)*** | **0.742 (0.681-0.804)*** | **0.710 (0.648-0.772)*** | 0.412 | 0.596 | 0.305 | 0.249 | 0.083 | 0.647 |
| N stage | N0 | | 391 | 193 | 198 | 0.780 (0.734-0.827) | 0.725 (0.675-0.775) | 0.654 (0.599-0.709) | 0.660 (0.606-0.714) | 0.058 | 0.559 | 0.095 | **0.040** | **<0.001** | 0.306 |
|  | N1 | | 203 | 193 | 10 | 0.888 (0.759-1.000) | 0.824 (0.662-0.985) | 0.773 (0.584-0.961) | 0.760 (0.569-0.950) | **0.002** | 0.191 | 0.885 | 0.895 | 0.746 | 0.801 |
| M stage | M0 | | 384 | 193 | 191 | 0.772 (0.724-0.820) | 0.715 (0.663-0.767) | 0.651 (0.596-0.706) | 0.650 (0.595-0.705) | 0.055 | 0.594 | 0.148 | **0.043** | **0.001** | 0.377 |
|  | M1 | | 210 | 193 | 17 | **0.941 (0.000-1.000)*** | **0.893 (0.791-0.995)*** | 0.757 (0.581-0.933) | **0.837 (0.730-0.945)*** | **<0.001** | 0.473 | 0.328 | 0.766 | **0.032** | 0.384 |
| BCLC stage | BCLC 0/A | | 290 | 193 | 97 | 0.691 (0.621-0.761) | 0.604 (0.530-0.679) | 0.476 (0.405-0.547) | 0.608 (0.538-0.678) | **0.021** | 0.198 | 0.253 | 0.507 | **0.001** | **0.039** |
|  | BCLC B/C/D | | 304 | 193 | 111 | **0.868 (0.832-0.926)*** | **0.839 (0.788-0.889)*** | **0.820 (0.766-0.874)*** | **0.715 (0.651-0.779)*** | 0.652 | **0.009** | 0.189 | **<0.001** | 0.091 | 0.213 |

* *p*-value < 0.05 versus each control group (top located in the category).

Bolded *p*-value is < 0.05 resulted from ROC comparison of the two markers in each subgroup.

LC, liver cirrhosis; HCC, hepatocellular carcinoma; HBV, hepatitis B virus; HCV, hepatitis C virus; ALT, alanine aminotransferase; UNL, upper normal limit; PVI, portal vein invasion; BCLC, Barcelona Clinic Liver Cancer; AFP, alpha-fetoprotein; PIVKA-II, protein induced by vitamin K absence; OPN, osteopontin; DKK-1, Dickkopf -1
